# Exploring Nurse and Patient Experiences of Developing Rapport During Oncology Ambulatory Care Videoconferencing Visits: Qualitative Descriptive Study

**DOI:** 10.2196/39920

**Published:** 2022-09-08

**Authors:** Paula D Koppel, Jennie C De Gagne, Sharron Docherty, Sophia Smith, Neil S Prose, Terri Jabaley

**Affiliations:** 1 School of Nursing Duke University Durham, NC United States; 2 Duke Cancer Institute Duke Health Durham, NC United States; 3 Department of Dermatology Duke University Durham, NC United States; 4 Department of Pediatrics Duke University Durham, NC United States; 5 Phyllis F Cantor Center for Research in Nursing & Patient Care Services Dana-Farber Cancer Institute Boston, MA United States

**Keywords:** clinician-patient relationship, nursing, oncology ambulatory care, rapport, telehealth, videoconferencing visits, videoconferencing, telemedicine, ambulatory care, cancer care, oncology nurse

## Abstract

**Background:**

Although videoconferencing between oncology patients and nurses became routine during the pandemic, little is known about the development of clinician-patient rapport in this care environment. Evidence that virtual visits may challenge nurses’ ability to form connections with patients, demonstrate empathy, and provide support suggests that videoconferencing may not ensure optimal care for persons with cancer. Establishing rapport during videoconferencing visits (VCVs) is important in oncology nursing, as rapport enables the nurse to provide emotional support and assistance to patients as they navigate their cancer journey.

**Objective:**

This study investigated the nature of nurse-patient rapport in ambulatory cancer care videoconferencing telehealth visits. Objectives included exploring (1) how patients with cancer and nurses describe experiences of and strategies for cultivating rapport and (2) similarities and differences between rapport in videoconferencing and in-person visits (IPVs).

**Methods:**

In this qualitative descriptive study, interviews were conducted from October 2021 to March 2022 with 22 participants, including patients with cancer (n=10, 45%) and oncology nurses (n=12, 55%), about their experiences of rapport building during VCVs. All interviews were analyzed using conventional content analysis. Data from nurses and patients were analyzed separately using identical procedures, with a comparative analysis of patient and nurse results performed in the final analysis.

**Results:**

Most patients in the study had experienced 3-5 video visits within the past 12 months (n=7, 70%). Half of the nurse participants (n=6, 50%) reported having participated in over 100 VCVs, and all had experiences with videoconferencing (ranging from 3 to 960 visits) over the past 12 months. In total, 3 themes and 6 categories were derived from the patient data, and 4 themes and 13 categories were derived from the nurse data. Comparisons of themes derived from participant interviews identified similarities in how nurses and patients described experiences of rapport during VCVs. Three themes fit the collective data: (1) person-centered and relationship-based care is valued and foundational to nurse-patient rapport in oncology ambulatory care regardless of how care is delivered, (2) adapting a bedside manner to facilitate rapport during VCVs is feasible, and (3) nurses and patients can work together to create person-centered options across the care trajectory to ensure quality care outcomes. Barriers to relationship building in VCVs included unexpected interruptions from others, breaks in the internet connection, concerns about privacy, and limitations associated with not being physically present.

**Conclusions:**

Person-centered and relationship-based approaches can be adapted to support nurse-patient rapport in VCVs, including forming a personal connection with the patient and using active listening techniques. Balancing the challenges and limitations with the benefits of videoconferencing is an essential competency requiring additional research and guidelines.

**International Registered Report Identifier (IRRID):**

RR2-10.2196/27940

## Introduction

### Background

During the COVID-19 global pandemic, the use of telehealth to provide oncology care as safely as possible increased dramatically. Some National Cancer Institute (NCI)–designated cancer centers reported that 33%-50% of all cancer patient encounters were telehealth visits, with utilization increases as high as 4693% compared to prepandemic rates [1]. Although telehealth includes a variety of technologies that support long-distance clinical health care, the use of videoconferencing visits (VCVs) as a modality to care for patients during the pandemic rapidly expanded in oncology during the pandemic [2]. Despite initial logistical challenges, many patients and oncology health care clinicians have decided that videoconferencing provides an innovative and effective way to receive and deliver care, yet little is known about how this modality of care affects patient-clinician relationships.

Review studies of videoconferencing in telehealth indicate that this computer-mediated modality has utility and comparable outcomes to in-person visits (IPVs) for patients with various chronic conditions [3-6]. Systematic reviews in oncology suggest that VCVs are feasible and can be effective for certain types of cancer care [7,8]. Prepandemic studies focused on videoconferencing for palliative care consultation and provision of support between patients, family caregivers, and community-based care clinicians [9-11] indicated that VCVs are often preferable for palliative care consultations [11,12], hospice family meetings [13,14], and support groups [15]. Recent studies suggest that patients are similarly satisfied with cancer care received via videoconferencing [16-18] and that oncology clinicians are increasingly receptive to videoconferencing and acknowledge its numerous benefits to patients [2].

### Knowledge Gaps

Although videoconferencing is feasible and can be effective for certain types of care, nurses, doctors, and mental health clinicians across health disciplines have expressed concern that the two-dimensional nature of videoconferencing interactions, including the loss of physical proximity, presence, and touch, depersonalizes and inhibits care and the clinician’s ability to understand the patient fully [19-22]. In palliative care studies, clinicians have indicated a reluctance to initiate emotional topics during VCVs as they cannot be physically present to provide necessary support [23,24] or ensure that patients have adequate privacy [14]. Suggestions for adapting in-person rapport techniques, such as small talk, eye contact, and body language, for VCVs have been discussed in the popular press and clinical commentaries [25-28]; however, little research has evaluated these modalities [29-32] or more advanced relational skills for providing presence [33-35], conveying caring [36] or empathy [37], and delivering person-centered care [38].

### Importance of Nurse-Patient Rapport

Rapport can be defined as a connection established with another person based on respect, acceptance, empathy, and a mutual commitment to engagement [39,40]. Interpersonal interventions that cultivate rapport between patients and clinicians can potentially improve patient health outcomes and satisfaction [41]. For persons with cancer, feeling personally known and connected with nurses and health care clinicians on a level beyond their disease process reduces suffering and improves satisfaction, health outcomes, and quality of life [42-45]. Research suggests that rapport facilitates a trusting and therapeutic relationship [46,47], enabling the clinician to become a source of emotional support for patients with cancer [39,48-50]. The literature supports that nurse-patient rapport facilitates the trusting relationship necessary to ensure holistic assessment of needs, personalization of care, and adaptive work.

### Critical Need for Research

COVID-19 necessitated a rapid adoption of VCVs into the standard care of patients with cancer, leaving little time to create thoughtful guidelines based on quality improvement or research focused on the impact of VCVs on patient-clinician relationships or care outcomes. Clinicians are seeking guidance regarding ways to transfer interpersonal skills to computer-mediated forms of care [51]. Studies comparing IPVs and VCVs indicate that (1) clinicians use less empathetic, supportive, and facilitating statements [37]; (2) exchange of information is reduced; and (3) patients present fewer problems [52] in VCVs; however, evidence regarding the effect of these findings on rapport and clinical care outcomes is lacking.

### Research Aim and Questions

The purpose of this qualitative descriptive study was to explore the experiences of nurses and patients participating in oncology telehealth VCVs, specifically in relation to the cultivation of rapport. This study explored rapport-building strategies as well as similarities and differences between rapport in VCVs compared to IPVs, with the aim of providing a foundation for relationship-building guidelines for telehealth videoconferencing.

## Methods

### Study Design

This qualitative descriptive design included interviews with patients with cancer and oncology nurses from October 2021 to March 2022. Semistructured interviews were conducted through the secure videoconferencing platform Zoom (Zoom Video Communications Inc). Participants were asked to share their general thoughts and feelings about VCVs; follow-up questions probed more deeply into their experiences of rapport during these visits. This descriptive qualitative approach, as described by Sandelowski [53,54], focused on experiences as directly reported by participants, while limiting deeper interpretation. This study included a sufficient number of nurse and patient participants to describe comprehensively the experiences of nurse-patient rapport in the new context of videoconferencing in ambulatory oncology [53,54], thus providing rich interpersonal experiences of VCVs. A brief description of the study components follows; details are available in the published protocol [55].

### Setting, Recruitment, and Eligibility Criteria

The study was conducted at an NCI-designated comprehensive cancer center in a northeastern metropolitan area of the United States. The scheduling and management of VCVs was centralized and standardized on the oncology center’s approved secure Zoom communication platform. As few VCVs occurred in the oncology center prior to COVID-19, in the winter of 2020, nurses were provided with tip sheets on the logistics of virtual visits and a recorded training session that included strategies for completing a physical examination virtually as well as suggestions for enhancing the “webside” manner (the manner in which a clinician interacts with a patient in VCVs) [26].

Purposive sampling was used to recruit participants who had recently used videoconferencing for their ambulatory care visits. Both patients with cancer and oncology nurses were included to enhance the understanding of how rapport building occurs within a nurse-patient dyad. Recruitment for the study began in September 2021. Nurses and patients were recruited remotely through a combination of efforts, including announcements at virtual nursing staff meetings and requests that participating nurses recommend colleagues and patients for the study. Efforts to recruit underrepresented participants were undertaken in the institution’s community-based satellite clinics. Patient recruitment focused on individuals who had nurse VCV teaching sessions or had seen their advanced practice nurse within the past 12 months. Participants were not compensated for their participation.

Patient inclusion criteria were (1) adult (18 years or older), (2) able to read and converse in English, (3) currently receiving care at the oncology institute, (4) current or former participant in at least 1 VCV with a nurse from the oncology center, and (5) enrolled in the oncology center’s patient portal. Patients with cognitive impairment or any condition that prohibited their ability to provide informed consent (eg, Alzheimer disease or related dementias) were excluded. Nurse inclusion criteria were (1) licensed registered or advanced practice nurse employed at the cancer institute for at least 1 year postorientation and (2) current or former participant in VCVs with patients at the oncology center. No restrictions on the length of nursing practice were necessary, as competency in developing rapport with patients is a foundational skill [56].

### Ethical Considerations

Approval was obtained from the Dana-Farber Cancer Institute Institutional Review Board (IRB) (protocol number: 21-318) and the Duke University Health System IRB for Clinical Investigations (protocol identification number: Pro00108787) prior to beginning the study. Written consent was obtained by the first author (PK) from all participants prior to the collection of any data.

### Data Collection Procedures

All interviews were conducted through the institution-approved platform by the first author (PK), a trained nurse scientist with 3 years of experience in qualitative methods. Although interviews were conducted in a videoconference format, only the audio portion was recorded. Audio recordings were transcribed verbatim. All identifiable participant information was removed, and records were assigned participant IDs to ensure confidential analysis of the text-based data.

Semistructured interviews were conducted. Participants were given an opportunity to tell complete stories about their experiences before the interviewer asked probing follow-up questions from a guide developed by the research team. Similar questions were asked of patient and nurse participants. The interview procedures and guide are published elsewhere [55]. By March 2022, the data collected were large enough to capture the rich experiences of nurses and patients but small enough to permit a thorough analysis [57]. The final interviews identified no new themes related to the research questions, indicating data saturation [58].

### Data Management and Analysis

Conventional content analysis was performed to analyze the narrative qualitative data, given how little is known to date and the consequent need for robust descriptive data. Codes were derived directly from the transcribed text data and kept close to participants’ descriptions [53,59]. Nurse and patient interviews were analyzed separately using identical procedures. Elo and Kyngas’ [56] content analysis management process was used to organize the analysis process into three phases: (1) preparation, (2) organization, and (3) reporting. This analysis process included creating, defining, and recording codes, categories, and themes and matching themes with exemplar quotations in a codebook using the data analysis management tool NVivo 12.0 (QSR International Pty Ltd). Initially, 2 research team members (authors PK and JDG) independently coded the same cases while compiling the codebook. Once agreement was reached on the codebook and coding of 20% of the transcripts, 1 team member (PK) completed coding the remaining transcripts while meeting with the team to discuss new codes and revise the codebook weekly. After coding 4 transcriptions from each group, the team decided to use 1 codebook to analyze nurse and patient data as the interview questions and responses were similar. Most of the codes were defined to capture both nurse and patient responses, but a small number were specific for each group. Although a common codebook was used, nurse and patient data were initially analyzed separately and then compared to explore similarities and differences. This process led to the discovery of a model to explain the overall research findings. Exemplar quotes from participants provide the evidence for our findings. Both individual and comparative analyses are reported to provide a rich and deep understanding of the data [56,59]. Findings are evaluated within the context of related theories, models, and evidence-based research. The study’s rigor, also described as trustworthiness in qualitative research [58,60], was enhanced by (1) conducting all analyses as a team with 18 combined years of qualitative research experience; (2) using weekly coding meetings to discuss and define all codes, categories, and themes; (3) collecting and analyzing the data concurrently [60]; (4) using detailed memos to create an audit trail of analytical decisions [58,61]; (5) confirming categories representing expansive and diverse experiences with exemplar quotations from multiple participants [58,62]; and (6) using member checking techniques by asking participants to clarify questions during interviews [58]. The consolidated criteria for reporting qualitative research (COREQ) checklist guided the reporting of results [63].

## Results

### Participant Characteristics

The NCI-designated comprehensive cancer center and satellite locations from where patients and nurses were recruited has an academic affiliation and Magnet Recognition for nursing excellence; per its primary care model, patients usually interact with the same nurse or advanced practice nurse. During the pandemic, the center used VCVs to follow patients during active treatment, provide education, and facilitate emotional support to online groups. The study sample included 22 participants (10 persons with cancer, 45%; 12 oncology nurses, 55%). Interviews with nurses lasted between 18 and 43 minutes and those with patients from 16 to 48 minutes. Persons with cancer were aged 36-67 years (mean 54.3, SD 8.31). The patient sample included more women (n=7, 70%) than men (n=3, 30%), and all participants identified as White/Caucasian. All patient participants had some college education, with half having graduate or professional degrees (n=5, 50%). Most participants were married (n=7, 70%), employed full-time (n=7, 70%), and had an annual income of US $100,000 or more (n=6, 60%). Patient participants were evenly split between those with a recent diagnosis and treatment of less than 1 year (n=5, 50%) and those having received treatment for over 1 year (n=5, 50%). Most patients had experienced 3-5 VCVs over the past 12 months (n=7, 70%) and at least 1 within the past 3 months (n=7, 70%). All patient participants reported spending at least 1 hour per day on their computers, with the majority spending 4 or more hours per day (n=7, 70%). Half of the participants reported having participated in at least 50 VCVs for work or personal connections within the past year (n=5, 50%). Multimedia Appendix 1 displays detailed characteristics of the patient participants.

Nurse participants (n=12, 55%) were mostly female (n=10, 83%) and identified as White/Caucasian (n=11, 92%). Their average age was 42.25 years (SD 8.62), with most aged either 31-40 years (n=5, 42%) or 41-50 years (n=5, 42%). All had attended at least some college, with the majority having a master’s degree (n=8, 67%). Nursing experience within the sample ranged from 4 to 35 years (mean 16.54, SD 8.67). Half of the participants had worked at the cancer center for over 10 years (n=6, 50%). Their experiences with VCVs ranged from less than 15 visits (n=5, 42%) to over 50 visits (n=3, 25%) within the past 3 months. Half of the nurse participants (n=6, 50%) reported having participated in over 100 VCVs within the past 12 months. Multimedia Appendix 2 displays detailed characteristics of the nurse participants.

### Qualitative Analysis of Patient Interviews

The data showed that patients view VCVs with their health care clinicians positively and appreciate having a personal relationship with their nurses, which they find is achievable in videoconferencing. Three key themes were identified during the analysis of patient interviews: (1) building rapport in VCVs and IPVs requires a personal touch, (2) rapport can facilitate trust in VCVs and impact how patients feel about their care, and (3) videoconferencing works well for some visits but is not ideal for others. Multimedia Appendix 3 displays a narrative description of each theme, associated categories, codes, and additional participant quotations.

#### Theme 1: Building Rapport in Videoconferencing Visits and In-Person Encounters Requires a Personal Touch

Theme 1 relates to the personal level, focusing on the patient’s description of the nurse’s (1) understanding of their life and feelings, apart from their diagnosis and treatment and (2) willingness to take time to present information and answer questions.

##### Being Known as a Person, Not a Patient

Some patients expressed that establishing rapport in videoconferencing is harder and that small talk at the beginning of the visit is more important than during IPVs. One participant said:

Things that help build the rapport would just be establishing the connection and relationship, like asking those little family questions and taking a moment to get a little bit beyond just the medical piece.

Patient 9 (P9)

They appreciated being asked questions about their families, occupation, and things that brought them joy and appreciated the nurses remembering these. One participant said the following:

Through conversations, my nurse remembered that my wedding anniversary was in May, my kid's birthday was coming up in July…you build that relationship, and…it makes it that much easier when you go through your treatment.

P5

One participant indicated that knowing their nurse on a personal level contributed to their sense that the nurse viewed them as a person, not just a patient. They appreciated the nurse’s willingness to share a few things about themselves and glimpses of the nurse’s personal environment when videoconferencing was done from the nurse’s home.

##### Being Heard and Knowing What Is Important

Participants expressed feeling heard, known, understood, validated, and valued when the nurse used active listening techniques. They had more confidence that the nurse understood what was uniquely important to them. One participant remarked:

A big part of rapport is that you want to feel heard and feel responded to in a way that acknowledges that you are heard.

P6

##### Taking Time to Provide Information and Answer Questions Thoughtfully

Participants felt the nurse was competent and cared about them as a person when they were given time to ask questions and received comprehensive, personalized answers. One participant noted:

I want to…ask as many questions as I need to, [without] a rush on the time…I don’t want a pat answer. I want it customized to me or to my situation.

P6

#### Theme 2: Rapport Can Facilitate Trust in Videoconferencing and Impact How Patients Feel About Their Care

This theme (1) focuses on how affect and nonverbal communication impact patients’ ability to develop rapport, trust, and confidence with their nurses during VCVs and (2) incorporates the outcomes that participants felt result from rapport, including enhanced comfort and healing.

##### Creating an Open Atmosphere With Positive Affect and Nonverbal Communication

Participants described the importance of the nurse having a friendly demeanor and using eye contact, facial expressions, vocal tones, and other types of body language to convey care, concern, attentiveness, and active listening. Participants felt these behaviors help establish rapport and set the tone in VCVs. One participant stated:

Facial cues help establish a good rapport. When you’re nodding your head, I realize that you are hearing me, understanding me, and maybe agree or at least sense my viewpoint.

P6

Participants felt the nurse’s ability to incorporate these behaviors and attributes influences the success of the VCV and encourages open engagement. One participant said:

A relationship…has to be two ways. The patient[’s]…willing[ness] to engage as well and be open [is] encouraged by the nurse.

P9

##### Building Confidence in the Plan of Care

Patients described how rapport and trust affect the sharing of information and build confidence. One patient explained:

If I don't have a good relationship with somebody, I don't feel comfortable enough to trust them, to tell them things, be open and honest.

P9

Patients described having confidence in their care when they felt their nurse understood what was important to them. One participant stated:

If you have a good rapport with them, you know that every decision they're making and everything they're doing is in your best interests.

P2

##### Promoting Comfort and Healing

Patients expressed that rapport with their nurses creates a sense of comfort. One participant indicated that rapport contributes to healing:

When you feel the [nurse] is looking at you in a compassionate way and caring about you as a human, it contributes to a positive outcome because you're taking in positive energy, and that's how it aids the healing process.

P6

One patient was equally enthusiastic about IPVs and VCVs and described in detail a seamless sense of knowing with their nurse:

My primary nurse, when she would see me, would have an intuition for how I was doing that particular day, and accommodate quickly, and that's because we'd built a rapport to know that. She could just say one thing to me, and I would know, “Oh, I've got to do this today,” because we had established the rapport, and I knew where she was coming from as well, so it allows for ease of the communication and a bidirectional understanding of what the needs are.

P4

#### Theme 3: Videoconferencing Works Well for Some Visits but Is Not Ideal for Others

The third theme focuses on the best circumstances for VCVs and IPVs. All the patient participants (1) expressed that VCVs are beneficial in certain situations, increase convenience, and improve access to clinicians and (2) hoped they would remain available post–COVID-19 for persons with cancer; however, they acknowledged that some oncology care needs to be provided in person.

##### Leveraging the Advantages of Videoconferencing Visits and Technology

Participants indicated that they find videoconferencing convenient and easy and that it saves time, money, and energy. One patient described the ease of reviewing test results with their nurse without disrupting their day:

Going over test results with me,…he's showing me the pictures. If you were in person, you'd have to try and peek around the computer…it really is a benefit, and then like 15 minutes are over, and I feel good about myself, and I can go on about my day. I don't need to get in a car and drive somewhere.

P5

Participants felt VCVs improve access to their nurses and other health care clinicians.

It allows me to go to the site that I want to for the labs and then I can do a video call for the appointment itself. So, it just gives me flexibility [and] more options.

P10

One participant indicated their nurse was able to be more attentive during their VCVs:

It's more relaxed, and people are under less of a time constraint. At the hospital…people seem more rushed.

P7

VCVs reduced the stress associated with travel to appointments, as 1 participant noted:

There's so many appointments [for which] you do have to actually be there in person, so to have some things that are taken off that, it really does reduce stress.

P8

Patients appreciated the ability to have supportive family members or friends participate in VCVs. Overall, the participants were willing to accept the challenges of a new technology to leverage its advantages.

##### Accepting the Challenges of a New Technology

Challenges included learning to use the technology, managing internet connectivity issues, and minimizing distractions. Older participants acknowledged their lack of confidence with videoconferencing technology but seemed empowered by learning to use it. One older participant noted:

People my age gets scared by the [idea of] technology…We’re getting better…every time it gets easier.

P7

Internet connectivity problems were acknowledged as challenges rather than barriers. One participant noted:

Losing the connection…That's the way of the world.

P7

Participants also mentioned the importance of managing distractions during VCVs. One participant stated:

If there's a lot of…distractions on both sides, you're not going to really feel like you're able to talk.

P3

All the patients interviewed recognized that some visits are not feasible in videoconferencing. One explained:

If you physically have to be there…[for] your cancer treatment, chemo, radiation, anything like that, or…an examination…you can't do some of those things over a video.

P5

Others favored an IPV when visualizing physical conditions was important:

In person, you can see the struggles physically that the person has. If they’re using a wheelchair. If they need assistance to go to the bathroom…but not virtually.

P1

Patient participants identified the type of information to be shared as another important determinant of VCV appropriateness. One patient stated:

I'm thinking if it was positive news, it's great on Zoom. Negative news might be not so great on Zoom. That might be…a little impersonal.

P9

Another participant indicated:

I would default to teleconferences over in person if everything else were equal…[an] exception might be if the decision or something you needed to learn was very complex.

P4

Another participant commented that they felt less comfortable sharing confidential matters in videoconferencing:

If you had something highly personal to discuss, I would feel less comfortable doing it virtually…we don't know how secure it is…[I]f I had problems that I didn't want to say out loud, I'd feel more comfortable saying it with [my] nurse in a little room…somebody else might also be listening.

P6

One participant had a difficult time describing why they preferred IPVs:

I don't know why [IPVs and VCVs are] different, but I suspect some of it has nothing to do with words or body language or anything [but] just wanting to be in the same room with the person.

P10

Of note, this participant had been receiving care over a long period, and videoconferencing meant they lost the support experienced by being in the cancer center and interacting with team members.

Although positive about VCVs, participants recognized the challenges of comprehending complex emotions and situations in their entirety in a virtual environment. They emphasized the importance of combining VCVs and IPVs:

You can build a good rapport through Zoom meetings, but it's important that there be face-to-face meetings intermingled. They don't have to be as often, but a combination of the two would be most beneficial.

P2

##### Finding Minimal Differences in Terms of Communication Effectiveness

Participants largely felt communication with their nurses in VCVs is comparable to that in IPVs. Most of the patients interviewed indicated that they felt comfortable expressing emotions and asking questions in VCVs. One participant expressed:

If that rapport is there, if [the nurse and patient are] paying attention to each other, it's just as good as an in-person visit.

P3

Another noted the following:

I didn't find there was much difference between the two…You could feel the concern. You could feel the genuineness in [the nurse’s] voice even though it was on the Zoom, which makes it so much easier to be able to deal with…a fresh diagnosis of cancer.

P2

However, some subtle aspects of communication during emotionally charged conversations may be compromised in videoconferencing. As 1 participant explained:

Communication [about scan results]…I feel like they're harder to do over video…It's sort of the contextual meaning of the words, if that makes sense…there's going to be a lot of words about the disease, and my question is, “How much time do I have?” That's really at the heart of it, right?... “What does this mean in terms of my quality of life and my quantity of life?” And nobody knows the answer to that, but the doctors and nurses…can give some contextual information. It's in the body language, it's in the tone of voice. It may not be in the words themselves.

P10

### Qualitative Analysis of Nurse Interviews

The data from nurse interviews demonstrated that rapport develops when nurses know their patients as persons and is achievable in VCVs with some adaptations. Four key themes were identified during the analysis of the nurse data: (1) rapport building begins with nurses knowing their patients as persons, (2) much of the bedside manner can be translated into the webside manner, (3) differences in videoconferencing that may impact rapport are important to recognize, and (4) cultivating nurse-patient rapport in VCVs is essential for quality care and nurse job satisfaction. Multimedia Appendix 4 displays each theme, associated categories, codes, and additional participant quotations.

#### Theme 1: Rapport Building Begins With Nurses Knowing Their Patients as Persons

This theme focuses on how nurses build rapport with their patients. Nurse participants described the importance of getting to know their patients holistically as individuals with needs, priorities, and lives apart from their cancer diagnosis. This connection facilitated trust and a sense of being well cared for that was valued by the nurse, the patient, and the patient’s family.

##### Viewing the Patient From a Holistic Perspective

Oncology nurses must be knowledgeable about their patients’ diagnoses and treatment plans and understand the impact of the disease on their quality of life. This holistic knowing was described by 1 participant:

We are here to talk about their health,…go through the review of systems and how they're feeling,…the labs,…the scan results, but I also like to say, “When you're not here, what are you doing outside of your cancer? Are you getting out of the house?” or “What's your family situation like? Are you working?” Just to know them on a more personal level and their families…the whole person, not just the cancer.

Nurse 10 (N10)

##### Attentiveness and Listening to Know the Patient and Understand Their Perspective

Nurses described how getting to know patients on a personal level creates a sense of bonding or rapport that facilitates understanding patient needs and allows the nurses to be empathetic and good patient advocates. One nurse participant explained:

Over the time that you spend with a patient in the clinic, you get to know them…their family, their likes, their dislikes, where they've traveled to. You make bonds. You might have similar situations or families. They tell you a lot of stories…I almost feel like I'm able to ask the physician a question on their behalf,…I try to be the patient advocate and put myself in the patient's shoes…It's like having a new friend that you just kind of learn everything about, and you want the best for them.

N6

##### Shared Experiences Enhance Trust, Open Communication, and Quality of Care

Nurses described intentionally asking patients questions about things they might have in common to facilitate a sense of connection. They often described rapport as foundational to building trust and open communication. One nurse noted:

Trust goes both ways. The patient has to trust the nurse to give them the correct care, and the nurse has to trust the patient to tell [them] when things are different or problematic. Some patients don't want to complain, and so they don't speak up when they have new pain or are more nauseous. If you have that rapport, you can kind of get to those underlying issues a little easier.

N2

Relationships built on rapport and trust were valued by nurses, in part because they believed this facilitates their ability to make good clinical decisions. One nurse stated:

When you connect with a patient, it’s easier to navigate their care because you understand them. You understand if they’re having certain symptoms, what it does and doesn’t mean. Rapport really helps in terms of clinical judgment.

N10

#### Theme 2: Much of the Bedside Manner Can Be Translated Into the Webside Manner

Theme 2 focuses on how nurses are adapting communication and relationship-building techniques to the videoconferencing setting, recently termed the “webside manner.” Most of the nurses felt some extra effort is necessary to enhance connection and ensure humanization during VCVs. Many expressed confidence in their ability to create and maintain strong nurse-patient relationships in VCVs, especially with patients they knew from IPVs.

##### Similar Ability to Communicate as In-Person Visits

Nurses viewed their communication with patients in VCVs comparable to in-person communication. One nurse practitioner stated:

The rapport remained heavily intact throughout videoconferencing. I didn’t feel I missed anything. Our conversations were very fruitful. We still had the same abilities to cover every topic.

N5

Some nurses even felt communication is enhanced as it allows them to schedule a VCV to share results or check on symptoms, which in the past might have been done by phone or email. One participant stated:

It actually improves our ability to communicate with patients. Oftentimes a patient I'm seeing virtually is not someone I would have brought into the office…so it allows a patient to communicate more with us than perhaps they would have otherwise.

N3

Another participant suggested that small adaptations to teaching are required for videoconferencing to be most effective:

If I'm doing a chemotherapy teach, I send them written instructions so that they can review them with me, and they can relay back. It helps if you communicate in multiple different ways at the same time.

N8

##### Making Extra Efforts to Connect

Many strategies mentioned for creating a comfortable environment applied to VCVs and IPVs. One nurse explained:

I make more of an effort to have small talk over video. I need to bring everyone in, calm everyone down, and make this seem as comfortable as possible before we get to what we need to talk about.

N2

Some nurses related a need to take extra measures to connect with patients on videoconferencing to avoid a dehumanizing effect. One participant expressed:

If you don't come off human in a virtual box, then it just becomes like an automated survey type of appointment. “Where are you experiencing pain? What is the pain now? Okay, are you taking medications?”

N12

Nurses described asking patients about their home environment, family, and important personal events and using humor. One participant described helping patients feel at ease:

[Videoconferencing is] more awkward than real life, but…you can kind of have a laugh over [it]: “Oh, this is so awkward, but isn't nice that we didn't have to go out in the cold today?” Just stating the obvious is one way to kind of break the ice.

N9

All the nurses described the importance of using eye contact and body language to demonstrate attentiveness and compassion. As 1 participant described:

[By] maintaining eye contact, nodding, trying to use hand gestures, trying to limit distractions so that I can really focus, I can be more outward with my emotions and…convey compassion and empathy with my face.

N3

Some nurses described being extra alert and attentive during VCVs to compensate for cues that might be more obvious in an IPV.

##### Ability to Form Relationships and Develop Rapport Remains Intact

Nurses reported an ability to build relationships and rapport over videoconferencing. One nurse stated:

We did the initial nursing assessment. We would talk about the emotional aspects, how patients were handling the diagnosis, how to start the treatments, and what that looked like. Overall, the rapport with the patients was not problematic.

N1

However, some nurses preferred IPVs for the initial visit and met patients in person whenever possible. One participant explained:

If it's a patient that I'm familiar with, it's a little easier [to establish rapport], but if it's a new patient that I've only met virtually during COVID, it's harder to build that rapport virtually, and if they're here to see the doctor and I can pop in, I try to pop in to meet them in person because I feel like it just adds to the rapport when you actually see them face to face.

N4

One benefit to video visits during COVID-19 was that nurses and patients could interact without wearing the face masks required for IPVs. Many nurses described the value of the unmasked interactions with patients afforded by VCVs. For example, a nurse noted:

I felt like we were able to develop that trusting relationship…Now that we are seeing people more regularly in person, some patients have actually said they appreciated the telehealth because I didn't have a mask on.

N7

#### Theme 3: Differences in Videoconferencing Visits That May Impact Rapport Are Important to Recognize

This theme focuses on how nurses described some unique characteristics of videoconferencing and their personal experiences of how videoconferencing creates barriers to or facilitates care.

##### There Is Only So Much You Can Do on Zoom

Nurses described clinical evaluations (eg, assessment techniques requiring hands-on physical examination) that were unfeasible in a VCV. As 1 nurse described:

There's no doubt about the fact that when you're in person, you know, you can put your hands on people. You can check an incision. You can listen to someone's lungs if they have a complaint. You can look in their mouth if they have a mouth sore. You can look at a rash.

N9

Participants considered some aspects of patient care, such as difficult or emotional conversations, best suited to IPVs. Nurses wanted to be with patients physically to evaluate their response and provide comfort and support. One nurse explained:

[What is lost in VCVs is] the body language and the cues that you would do in person. If you're having a difficult conversation, I would roll my chair over and bridge the gap physically…[During] a hospice talk, I might put a hand on a shoulder or on an arm or on a knee, offer tissues…show [in] nonverbal ways that I care, I'm concerned, and I'm really listening and engaged. I feel like you can only do so much on a Zoom [call].

N4

For many of the nurses, not being in the same physical space as the patient inhibited their ability to assess the encounter (eg, how the patient received information) thoroughly. The abrupt endings of video visits made some participants uncomfortable. One nurse participant explained:

Sometimes I leave a virtual visit not knowing how things landed with the patient because I'm not there, so if it's a big conversation, or a difficult conversation, or we're talking about a lot of things at once, sometimes it's harder to read the patient and how things are being processed. Whereas in person, before I leave a room, I usually make sure that things [are okay].

N3

##### There’s No Place Like Home

Nurses observed that patients seem more comfortable in their own homes during VCVs and appreciated the opportunity to see into the patients’ home environment. As 1 nurse stated:

[Patients] may feel they have more time to answer questions when they're on the computer in their own environment and they’re not stressed about coming into an appointment in an unfamiliar situation.

N6

##### Barriers Related to Technology, Language, and Hearing

Nurses noted that the patient’s level of experience with videoconferencing technology could present a barrier, and they had mixed impressions about its impact on rapport building. One nurse noted:

Patients have different levels of comfort and knowledge about the technology. Some…can't figure it out. Sometimes those barriers, like not being able to see them or make the eye contact, diminish rapport.

N4

Some nurses acknowledged difficulties but felt they do not interfere with rapport building:

If the Wi-Fi’s bad or the connection’s bad or…they can’t hear me or I can’t hear them, or if they’re trying to bring up audio or the video. I think those can impact [the patient’s stress level], but I’d say for the most part, I don’t think I’ve had difficulty developing rapport with patients.

N7

Videoconferencing with patients who have hearing impairments or do not speak English presented an additional challenge according to some nurse participants. One nurse noted:

We have taken care of [some]ethnically diverse patients lately, and I find that's definitely more challenging on video because they're often not sitting in front of me. There could be a language barrier or they're just not quite understanding what you're trying to communicate. That definitely takes extra work, [and] you have to be sensitive to those things.

N11

#### Theme 4: Cultivating Nurse-Patient Rapport in Videoconferencing Is Essential for Quality Care and Nurse Job Satisfaction

This theme focuses on practice adaptations to accommodate the addition of videoconferencing as a modality of care. Nurses expressed a desire for guidelines related to balancing patient convenience with the clinician’s need to accomplish clinical goals. Maintaining a sense of connection or rapport with patients was described as important in ensuring quality care and the nurse’s sense of fulfillment.

##### Everyone Is Still Learning, but We Are Finding Ways to Adapt

Nurses acknowledged that using videoconferencing requires them to adapt, and some expressed mixed feelings. Overall, participants’ descriptions suggested that they and their patients are acclimating. One participant said:

The pandemic taught us a lot…In health care at least, we learned there's a lot of things we can do, and how quickly we all acclimated is amazing, and so I just hope [VCVs] stay around.

N5

Another nurse described their experience of adapting to VCVs more tentatively:

It's kind of bumpy…I do prefer an in-person visit…but a telehealth visit with video is a lot better than just the telephone…It's still new, so I have mixed feelings about it.

N6

##### Determining What Is Best for the Person and the Patient

Nurses recognized the advantages and limitations of videoconferencing, expressing that the best option differs from patient to patient and nurse to nurse, even for difficult conversations. Nurses commonly described a preference for sharing bad news with patients in person. One participant shared:

Someone asked me today, “Am I going to die?” and I moved closer to her…these moments of eye contact where you really have to make a connection with someone so that they know you're telling them the truth, that's hard over Zoom.

N9

However, 1 nurse described that patients and nurses are acclimating:

When we were at the beginning of the pandemic, we were worried that having a hospice conversation would be really hard over video…[but] if the patient's not feeling well, they don't want to come in. They have their family there,…the world is changing, and people are more and more used to having hard conversations over Zoom.

N10

Nurses reported a need to balance patient preferences with clinical evaluation, communication, and rapport factors. Some felt patients are the major beneficiaries of videoconferencing, with clinicians left to figure out how to adapt. Participants did view videoconferencing as an opportunity to follow patients more closely. Nurses felt that granting patients a choice over the format of visits is a means of empowering them.

The nurses interviewed hoped that VCVs would continue to be an option for seeing patients after the pandemic. As 1 nurse stated:

I hope...[for] sort of a hybrid where we can connect with our patients virtually, and…in person. We can be there in multiple ways, but always constantly there.

N8

##### The Unique Challenges Presented by Videoconferencing Require Professional Development and Practice Guidelines

The collective experiences of the nurses in the study revealed a need and desire for practice guidelines for nurses and patients and professional development. As 1 nurse explained:

It is important to have guidelines…on an institutional level,…for patients to understand that it isn't the sole way that we can take care of them, . . . they do need to still see someone in person, because [otherwise] that could be dangerous. Things can be missed, so having those guidelines from an institutional level would…set the boundaries and the safety checkpoints to ensure that we're providing the best care we can for patients and in proper intervals.

N5

Several nurse participants expressed disappointment that as the pandemic waned, they were encouraged to bring patients to the clinic for IPVs when a VCV would have been sufficient. One nurse stated:

I think that [VCVs] serve a purpose for a very specific group of our patients…I'm not sure why it took the pandemic for us to realize that they were a really useful and appropriate way [to provide care]...They've been incredibly successful for providers and for patients, in certain settings, and I wish that we could use them more. I was kind of expecting a revolution to happen, and I'm really disappointed [this has not] played out.

N9

##### Developing Rapport With Patients Is Important to Oncology Nurses

Without exception, the nurses interviewed described rapport as important to nursing practice and essential to high-quality care. Nurses described a sense of professional satisfaction from developing close relationships with their patients. As 1 nurse stated:

[Rapport] really can make my day. It's one of the best parts of the job, and that's probably what brings people to this line of work…It's a job with a lot of stress and it can be emotionally taxing…[H]aving that level of satisfaction and warmth and looking forward to seeing our patients really makes the job so enjoyable. It's very helpful in…grounding me in what I do and why I do it every day.

N5

##### Comparison of Patient and Nurse Analyses

Comparison of the themes derived from the interviews showed similarities in how the nurses and patients described the experience of rapport during VCVs. Taken in totality, the data from all participants demonstrated that a person-centered and relationship-based approach can support nurse-patient rapport and the development of guidelines in videoconferencing for persons with cancer. Three themes fit the collective data: (1) person-centered and relationship-based care is valued and foundational to nurse-patient rapport in oncology ambulatory care regardless of how care is delivered, (2) adapting a bedside manner to facilitate rapport in VCVs is feasible, and (3) nurses and patients can work together to create options across the care trajectory that are person-centered and ensure quality care outcomes.

#### Theme 1: Person-Centered and Relationship-Based Care Is Valued and Foundational to Nurse-Patient Rapport in Oncology Ambulatory Care Regardless of How Care Is Delivered

This theme focuses on the importance of person-centered and relationship-based care to both the nurse and the patient. The synergy between the patient’s desire to be known on a personal level and the nurse’s desire to know the patient provides a foundation for rapport building in oncology care delivered in an ambulatory care setting in both IPVs and VCVs. Patients and nurses used the words “treated as a person not just a patient” to describe a desire for holistic and personalized knowing. One patient emphasized the importance of the nurse and patient being authentic:

It's about me being a person and the nurse being a person rather than a job and a patient. You can't build rapport around a job and a patient. That's not doable, so anything that makes the nurse more of a person…She asks how things are going. I ask how things are going, and she answers…that little bit of back and forth makes a big difference…I trust a person, and I feel more seen as a person.

P10

Nurses and patients emphasized the importance of the nurse-patient relationship being built on mutual trust and understanding. This evolved out of knowing each other and the shared experience of managing a cancer diagnosis. One patient noted:

[Rapport is] having developed a relationship of trust and understanding…like you’re on the same page. You understand each other…It’s very important to have rapport because there are times when you really need somebody who understands what you’re going through…so [you]’re not feeling alone and isolated.

P1

#### Theme 2: Adapting a Bedside Manner to Facilitate Rapport in Videoconferencing Visits Is Feasible

This theme focuses on common strategies identified by patients and nurses to facilitate rapport and communication during VCVs. The behaviors nurses and patients described as important were the same for VCVs and IPVs; however, both nurses and patients seemed acutely aware of how body language in particular communicates attentiveness, compassion, and empathy in videoconferencing. One nurse described the importance of “maintaining eye contact, nodding, trying to use like hand gestures while I'm talking, trying to limit distractions in the visit so that I can really focus and…convey compassion and empathy with my face” [N3].

Most of the participants expressed that cultivating rapport and communicating in a VCV is feasible but that being able to have at least some IPVs is best for nurturing the nurse-patient relationship. As 1 nurse stated:

We see a lot of our patients frequently, and the ability to be able to alternate visits, like every other or every second visit in person and do the other ones on video has been a great benefit.

N7

#### Theme 3: Nurses and Patients Can Work Together to Create Options Across the Care Trajectory That Are Person-Centered and Ensure Quality Care Outcomes

This theme highlights how both patients and nurses recognized that not all care can be provided in VCVs but that, when used selectively, good care can be provided in VCVs. As 1 nurse stated:

Patients [who] are established and they're just coming in for a scan review or a quick lab check, it just makes their life so much easier. You already have an established relationship, so it doesn't change anything.

N10

Similarly, a patient stated:

I find it's a really good way for communicating…[F]or certain visits, it really works.

P10

## Discussion

### Principal Findings

The purpose of this qualitative study was to describe the experiences of nurses and patients participating in oncology telehealth VCVs, with a focus on their ability to cultivate rapport. The findings suggest that rapport building is achievable within VCVs, with many traditional bedside, in-person strategies transferrable to the videoconferencing environment. There was a striking similarity in the descriptions of rapport building by patients and oncology nurses; both described a personal connection as foundational to building a trusting relationship and important for high-quality, satisfying care. Patients and nurses acknowledged that videoconferencing has benefits, challenges, and limitations but were interested in determining when and how to make optimal use of this new care modality.

Few studies to date have focused on ambulatory oncology nurse-patient rapport in VCVs. Consistent with previous research on relationship building in oncology IPVs [42-45], all participants in our study described the importance of the patient being known as a person rather than solely as a patient. Nurses described strategies to know the patient more holistically, and some patients appreciated knowing the nurse in a more personal way. Nurses described using self-disclosure, a common strategy for building relationships in nursing practice [64], to create a personal connection with their patients. Self-disclosure has been described as beneficial in building rapport with persons who have cancer [44,65].

Patients in our study valued being heard and appreciated the nurse taking time to provide information and answer their questions thoughtfully, and nurse participants described the importance of being attentive and listening closely. The importance of these behaviors is well described in the literature [39,44,48-50,66]. In a recent study exploring nursing listening behaviors, 70% of patients described nurse eye contact and attentiveness as an indication that the nurse was listening [66]. Conversing with patients on a personal level by asking and answering questions and providing a welcoming environment was described as a way to “transform an otherwise inauspicious moment into a powerful connection” [66]. Studies in oncology ambulatory care note that the absence of these caring behaviors leads patients to describe care as dehumanizing [67,68].

Our findings reflect attributes used to define rapport in the literature, including a shared experience comprising positive affect; mutual respect, acceptance, care, and concern; and behavioral synchrony [39,40,69]. Patient and nurse participants indicated that a shared connection facilitates outcomes similar to those described in the literature, including trust [46,66], open communication [39], comfort [66,70], confidence in the plan of care, and improved clinical judgment. Studies have suggested that rapport not only influences the patient’s perception of care but also has a tangible impact on care outcomes [41,71].

Both nurses and patients in our study felt that many of the strategies that build rapport in IPVs are effective in VCVs. Similarly, Elliott et al [72] suggested that patients who are satisfied with telemedicine encounters value their relational experience. The terms associated with their study’s code for “build rapport” align with the findings in our study, including “affective connection/comments of appreciation, trust-building, caring, concerned bedside manner, used nonverbal gestures that show care and concern, provided emotional support, understanding, developed a partnership, helpful, nice, friendly, easy to talk to” [72]. Other studies of videoconferencing have described the importance of knowing the patient as a person [73] and using eye contact, facial expressions [74,75], and other body language [76] in conveying attentiveness and emotions, including empathy. Our participants described the importance of nonverbal behaviors during VCVs to enhance communication and connection.

Although limited studies have explored relationship building in oncology VCVs, our study had findings similar to a qualitative study by van Gurp [24], which involved interviewing patients receiving care via videoconferencing with a palliative care team of whom over half were nurses. This study found that personalized patient-clinician relationships during videoconferencing are facilitated by consistent, empathetic engagement with the same clinician, and like our study, relationships were facilitated by clinicians who listened attentively and exchanged a mixture of medical and personal information during conversations.

Other nursing studies have described technology failures as a barrier to rapport [33,77]. Some participants in our study noted that disruptions in the home environment or internet connectivity have the potential to interfere with rapport, although this was not a significant concern. One patient participant expressed a preference to share confidential information with their nurse in person, due to uncertainty about videoconferencing security. In van Gurp’s [24] study, the ability to see but not touch made some palliative care clinicians reluctant to share difficult news (eg, a nurse expressed a desire to be able to provide physical comfort in these situations); however, for some patients, this physical separation made it easier for them to express feelings. These different perspectives were articulated by nurses and patients in our study. Some participants felt being in the comfort of their own homes made sharing emotions and concerns easier, while others felt it was important to have difficult news shared in person.

Although van Gurp’s study [24] was the only research we identified that focused on rapport and videoconferencing in an oncology population, incidental findings from other nursing studies prepandemic suggest that setting up the environment to allow mutual attentiveness (eg, adjusting the camera position and audio volume) and ensuring privacy facilitate rapport in video visits [9,14,24,33,78-81]. Other studies show that a nurse’s positive attitude toward technology [78] and the availability of others to assist patients onsite are helpful [33,78,82-84]. In our study, nurses and patients seemed equally willing to accept the challenges associated with videoconferencing, given the perceived benefits. There were relatively few concerns expressed about using the technology or its impact on relationship building. This phenomenon may be due to the increased need for and utilization of videoconferencing during the pandemic, especially for persons with cancer. In a study using videoconferencing with older adults experiencing depression, patients described the technical challenges as “little things,” which the authors suggested was because the patients benefited from videoconferencing in many ways [85]. A central finding of this study was that an optimistic outlook on videoconferencing influenced the expectations and attitudes of both patient and clinician participants, mitigating negative feelings about technological challenges [85].

Concerns that videoconferencing would depersonalize care [19-22] were not supported by our findings. Although patients and nurses expressed that IPVs are more desirable in certain situations, none of the participants described their VCVs as impersonal; on the contrary, many described their ability to develop rapport and communicate effectively during VCVs. According to Barrett’s [33] ground theory, the primary function of the nurse in videoconferencing is to provide an operational, clinical, therapeutic, and social presence. The nurse cultivates rapport in the nurse-patient relationship during videoconferencing by providing reassurance and support (therapeutic presence) and creating connection, thus “giving the patient the sense they are ‘in the room’ with them” (social presence) [33]. Although the word “presence” was not used to describe behavior or attributes by any of our participants, the need to have more focused attention was described by several of the nurses and 1 patient. The importance of telepresence, generally described as feeling physically present during a computer-mediated encounter [86], has been the focus of some research [21,33,36].

Our findings suggest that oncology nurses and persons with cancer are receptive to integrating videoconferencing into the care trajectory but feel it is important to determine what type of visit will best serve the patient for each care encounter. This like-mindedness bodes well for nurse-patient collaboration on decisions about whether a visit should occur virtually or in person at the cancer center. Similar to our results, recent studies indicate that both patients and clinicians are increasingly receptive to oncology care provision through VCVs and want the option of videoconferencing to extend post–COVID-19 [87,88]. As in previous nursing studies, both patients and nurses in our study acknowledged that video visits could not replace all in-person encounters [24,33,79,83,85,89]. This was felt to be especially true for serious conversations [14,24] and initial encounters [83,85]. At least 1 nurse in the study looked for opportunities to physically see patients during their chemotherapy treatments whom they had only met in VCVs in order to enhance their connection. Although the study did not ask patients during the interview about how long they had known their nurses, 50% of the patient participants had been in treatment for less than a year. Most, though not all, patients and nurses in the study, felt IPVs visits are better for more difficult conversations. Our findings and those of previous studies suggest that decisions about whether to use videoconferencing must consider the patient’s preferences and everchanging needs. This conclusion is supported by the Institute for Healthcare Improvement’s (IHI) recent white paper on telemedicine [90], leading some to suggest that decisions regarding virtual care require the following amendment to the precision medicine maxim: “Provide the right treatment, to the right patient, at the right time, and in the right place” [91].

### Implications for Practice, Policy, and Professional Development

We recommend that policy and practice guidelines be person centered, allowing clinicians to assess in real time the type of visit that can best meet the patient’s holistic needs. The IHI has proposed a framework for telemedicine that is safe, equitable, and person centered: “Honoring the patient’s wishes as long as those desires are consistent with delivering safe and effective care” [90]. Our results suggest that nurses and persons with cancer have an appreciation for the challenges as well as the benefits of VCVs and understand that this environment may not always provide the best option for high-quality, safe care.

Although the pandemic forced a rapid adoption of video visits, clinicians remain uncertain about how best to provide virtual care. In a recent mixed methods study by Elsevier Health of 3000 nurses and doctors, over half of the clinicians felt telehealth would negatively impact their ability to demonstrate empathy and requested guidance on learning webside skills [51]. Although most of our participants felt able to transfer many bedside skills into VCVs, there is mixed evidence in the literature on whether videoconferencing can produce the same empathetic experience as an IPV [92]. Notable efforts have been made to provide guidance on a webside manner [26-28,93,94], but additional research is needed to ensure that professional development and practice guidelines are evidence based, and our findings provide data regarding relationship-based care and patient-centered communication in a videoconferencing environment. Schools of nursing and medicine need to prepare future practitioners to care for patients in a virtual health care environment, with competencies specific to these digital tools [92,95]. Fortunately, nursing theories [22,33,35] and conceptual models [96] exist to support this important work, and relationship-based care has been successfully incorporated into health care clinician curriculum and professional practice models [97,98], showing improvement in health care delivery [41,99,100].

Our findings identified barriers to rapport building in videoconferencing that have been previously identified in studies on videoconferencing, including interruptions due to breaks in the internet connection [32,33,77,85,90], concerns about privacy, [9,12,24,83], and the limitations imposed by a lack of physical presence [24,32,33,83,90]. Nurses and patients in our study recognized that balancing these challenges and limitations with the benefits of videoconferencing is an essential competency requiring additional research and guidelines.

### Future Research

Research is needed to better understand how specific rapport-building strategies can be translated or adapted to VCVs, including nonverbal communication and active listening techniques. Additionally, exploring how various types of visits (eg, first encounters, delivery of bad news) and contextual factors (eg, virtual backgrounds, quality of connectivity, sensory and language barriers) influence rapport building in videoconferencing would be useful.

Ensuring adequate access to VCVs is multifaceted and requires not only that adequate devices and Wi-Fi resources be available and affordable but also that patients and providers have the skills and support to incorporate them into their care services [87,101]. Investigating how digital literacy and access to telehealth technology influence patient and nurse utilization of VCVs, while not addressed in this study, must be a focus of future studies if videoconferencing is to become a mainstay of health care. Existing health and technological disparities became more apparent during COVID-19 [101,102], and future studies are needed to better understand the relationship between the services offered and the needs and abilities of patients and providers. For example, the lack of access to adequate devices and Wi-Fi resources for VCVs is well documented [1,103,104], but the impact of other factors, such as language barriers and availability of privacy and safe spaces for VCVs [105], require better understanding and accommodation. Specifically in relationship to rapport, there is some evidence that qualitative differences between type of device, strength of broadband, and level of literacy impact the level and quality of empathetic communication within VCVs [92]. Addressing these factors is essential if we are to avoid increasing the digital divide among populations who may already be at a higher risk for cancer due to social and economic disparities [106,107]. Infusing a health equity lens as we generate new knowledge about VCVs can help prevent the augmentation of health disparities and promote health and scientific equity, especially for groups that are underrepresented.

Other interesting questions generated from our findings include the role of nurse self-disclosure on the development of rapport and whether videoconferencing can create a safer space or reduce the power differential between clinicians and patients. Our study shows the importance of rapport in the nurse-patient relationship and its impact on patient care and nursing job satisfaction, highlighting the need for additional research in this area.

### Strengths and Limitations

The strengths of this study include the collection of data from persons with cancer and oncology nurses. Multiple strategies were used to improve the trustworthiness of findings. The analysis included categories covering a wide range of participants’ responses to achieve credibility. Dependability was ensured by selecting quotations from multiple participants and identifying how these were linked to results. To allow the findings to be transferred or applied to other settings or groups, the context of the study and participant characteristics were thoroughly described.

This study has several limitations. First, the data analysis and its interpretation depended on the researchers’ skills, assumptions, and experience. Second, data were collected from 1 cancer center and were therefore influenced by the organizational system and its practices. Third, to minimize the burden on the participants, member checking was not used after the data were analyzed; however, the researcher conducting the interviews frequently validated their understanding of participants’ answers during the interview. Fourth, despite our efforts to include nurses and patients who might otherwise have been underrepresented in the study, the study participants mostly self-identified as female, were White/Caucasian, and were employed full-time, with at least some college education. There was a wide range of experience with VCVs among the participants; however, most were using computer technology and videoconferencing for work and personal affairs, suggesting their technological skills, devices, and internet access might not be representative of most patients with cancer and their nurses. This limits the ability to generalize the study findings to patients and providers with less access to and experience with VCV technology.

### Conclusion

Although providing care within the videoconferencing environment may require adapting practices, the essential nature of nursing need not be affected. In this study, the overall synergy between the nurse and patient data and the specific descriptions from patients and nurses on ways to establish rapport were striking. Patients and nurses considered rapport essential to the nurse-patient relationship and high-quality care, thus affirming nursing’s commitment to person-centered care and the profession’s capacity to understand patient needs holistically. Nurses are well positioned to assume an advocacy role for care across the cancer continuum and leadership in the development of guidelines, policies, and future research inquiry on VCVs. Contrary to concerns that videoconferencing would be impersonal and inhibit rapport and relationship building, this study indicated that rapport can be established during VCVs and that many of the strategies used during IPVs are equally successful in videoconferencing. Findings from this study provide some of the descriptive research necessary for the development of evidence-based practice guidelines and interventions to support nurse-patient therapeutic relationships during VCVs.

## Appendix

Multimedia Appendix 1 Characteristics of patient participants.

Multimedia Appendix 2 Characteristics of oncology nurse participants.

Multimedia Appendix 3 Patient data analysis.

Multimedia Appendix 4 Nurse data analysis.

## Abbreviations

IHC: Institute for Healthcare Improvement
IPV: in-person visit
IRB: institutional review board
NCI: National Cancer Institute
VCV: videoconferencing visit

## References

[1] Knudsen KE, Willman C, Winn R (2021). Optimizing the use of telemedicine in oncology care: postpandemic opportunities. Clin Cancer Res.

[2] Alpert JM, Taylor G, Hampton CN, Paige S, Markham MJ, Bylund CL (2022). Clinicians' perceptions of the benefits and challenges of teleoncology as experienced through the COVID-19 pandemic: qualitative study. JMIR Cancer.

[3] Almathami HKY, Win KT, Vlahu-Gjorgievska E (2020). Barriers and facilitators that influence telemedicine-based, real-time, online consultation at patients' homes: systematic literature review. J Med Internet Res.

[4] Ignatowicz A, Atherton H, Bernstein CJ, Bryce C, Court R, Sturt J, Griffiths F (2019). Internet videoconferencing for patient-clinician consultations in long-term conditions: a review of reviews and applications in line with guidelines and recommendations. Digit Health.

[5] Penny RA, Bradford NK, Langbecker D (2018). Registered nurse and midwife experiences of using videoconferencing in practice: a systematic review of qualitative studies. J Clin Nurs.

[6] Speyer R, Denman D, Wilkes-Gillan S, Chen Y, Bogaardt H, Kim J, Heckathorn D, Cordier R (2018). Effects of telehealth by allied health professionals and nurses in rural and remote areas: a systematic review and meta-analysis. J Rehabil Med.

[7] Kitamura C, Zurawel-Balaura L, Wong RK (2010). How effective is video consultation in clinical oncology? A systematic review. Curr Oncol.

[8] McGrowder DA, Miller FG, Vaz K, Anderson Cross M, Anderson-Jackson L, Bryan S, Latore L, Thompson R, Lowe D, McFarlane SR, Dilworth L (2021). The utilization and benefits of telehealth services by health care professionals managing breast cancer patients during the COVID-19 pandemic. Healthcare (Basel).

[9] Funderskov KF, Boe Danbjørg D, Jess M, Munk L, Olsen Zwisler A, Dieperink KB (2019). Telemedicine in specialised palliative care: healthcare professionals' and their perspectives on video consultations-A qualitative study. J Clin Nurs.

[10] Jess M, Timm H, Dieperink KB (2019). Video consultations in palliative care: a systematic integrative review. Palliat Med.

[11] Steindal SA, Nes AAG, Godskesen TE, Dihle A, Lind S, Winger A, Klarare A (2020). Patients' experiences of telehealth in palliative home care: scoping review. J Med Internet Res.

[12] Funderskov KF, Raunkiær M, Danbjørg DB, Zwisler A, Munk L, Jess M, Dieperink KB (2019). Experiences with video consultations in specialized palliative home-care: qualitative study of patient and relative perspectives. J Med Internet Res.

[13] Bradford N, Young J, Armfield NR, Bensink ME, Pedersen L, Herbert A, Smith AC (2012). A pilot study of the effectiveness of home teleconsultations in paediatric palliative care. J Telemed Telecare.

[14] Oliver D, Washington K, Wittenberg-Lyles E, Demiris G, Porock D (2009). 'They're part of the team': participant evaluation of the ACTIVE intervention. Palliat Med.

[15] Melton L, Brewer B, Kolva E, Joshi T, Bunch M (2016). Increasing access to care for young adults with cancer: results of a quality-improvement project using a novel telemedicine approach to supportive group psychotherapy. Pall Supp Care.

[16] Nordtug B, Brataas HV, Rygg LO (2021). Patient experiences with videoconferencing as social contact and in follow-up from oncology nurses in primary health care. Health Psychol Open.

[17] McCabe HM, Smrke A, Cowie F, White J, Chong P, Lo S, Mahendra A, Gupta S, Ferguson M, Boddie D, Mmekka W, Stirling L, Campbell L, Jones RL, Nixon I (2021). What matters to us: impact of telemedicine during the pandemic in the care of patients with sarcoma across Scotland. JCO Global Oncol.

[18] Calton B, Shibley WP, Cohen E, Pantilat SZ, Rabow MW, O'Riordan DL, Bischoff KE (2020). Patient and caregiver experience with outpatient palliative care telemedicine visits. Palliat Med Rep.

[19] Brahnam S (2017). Comparison of in-person and screen-based analysis using communication models: a first step toward the psychoanalysis of telecommunications and its noise. Psychoanal Perspect.

[20] Chhabra AM, Chowdhary M, Choi JI, Hasan S, Press RH, Simone CB (2021). A national survey of radiation oncology experiences completing tele-consultations during the coronavirus disease (COVID-19) pandemic. Adv Radiat Oncol.

[21] Grumme V, Barry C, Gordon S, Ray M (2016). On virtual presence. Adv Nurs Sci.

[22] Locsin RC, Purnell M (2015). Advancing the theory of technological competency as caring in nursing: the universal technological domain. Int J Hum Caring.

[23] Lally K, Kematick BS, Gorman D, Tulsky J (2021). Rapid conversion of a palliative care outpatient clinic to telehealth. JCO Oncol Pract.

[24] van Gurp J, van Selm M, Vissers K, van Leeuwen E, Hasselaar J (2015). How outpatient palliative care teleconsultation facilitates empathic patient-professional relationships: a qualitative study. PLoS One.

[25] Car J, Koh GC, Foong PS, Wang CJ (2020). Video consultations in primary and specialist care during the covid-19 pandemic and beyond. BMJ.

[26] Chua IS, Jackson V, Kamdar M (2020). Webside manner during the COVID-19 pandemic: maintaining human connection during virtual visits. J Palliat Med.

[27] Webb M, Hurley SL, Gentry J, Brown M, Ayoub C (2021). Best practices for using telehealth in hospice and palliative care. J Hosp Palliat Nurs.

[28] Banerjee SC, Staley JM, Howell F, Malling C, Moreno A, Kotsen C, Parikh D, Parker PA (2021). Communicating effectively via tele-oncology (Comskil TeleOnc): a guide for best practices for communication skills in virtual cancer care. J Cancer Educ.

[29] Dalley D, Rahman R, Ivaldi A (2021). Health care professionals' and patients' management of the interactional practices in telemedicine videoconferencing: a conversation analytic and discursive systematic review. Qual Health Res.

[30] Henry B, Ames L, Block D, Vozenilek J (2018). Experienced practitioners' views on interpersonal skills in telehealth delivery. Internet J Allied Health Sci Pract.

[31] Newcomb AB, Duval M, Bachman SL, Mohess D, Dort J, Kapadia MR (2021). Building rapport and earning the surgical patient's trust in the era of social distancing: teaching patient-centered communication during video conference encounters to medical students. J Surg Educ.

[32] Shaw SE, Seuren LM, Wherton J, Cameron D, A'Court C, Vijayaraghavan S, Morris J, Bhattacharya S, Greenhalgh T (2020). Video consultations between patients and clinicians in diabetes, cancer, and heart failure services: linguistic ethnographic study of video-mediated interaction. J Med Internet Res.

[33] Barrett D (2017). Rethinking presence: a grounded theory of nurses and teleconsultation. J Clin Nurs.

[34] Tuxbury J (2013). The experience of presence among telehealth nurses. J Nurs Res.

[35] Groom LL, Brody AA, Squires AP (2021). Defining telepresence as experienced in telehealth encounters: a dimensional analysis. J Nurs Scholarsh.

[36] Sitzman, K (2017). Evolution of Watson’s human caring science in the digital age. Int J Hum Caring.

[37] Liu X, Sawada Y, Takizawa T, Sato H, Sato M, Sakamoto H, Utsugi T, Sato K, Sumino H, Okamura S, Sakamaki T (2007). Doctor-patient communication: a comparison between telemedicine consultation and face-to-face consultation. Intern Med.

[38] Heckemann B, Wolf A, Ali L, Sonntag SM, Ekman I (2016). Discovering untapped relationship potential with patients in telehealth: a qualitative interview study. BMJ Open.

[39] Epstein R, Street R (2007). Patient-Centered Communication in Cancer Care: Promoting Healing and Reducing Suffering.

[40] Tickle-Degnen L, Rosenthal R (1990). The nature of rapport and its nonverbal correlates. Psychol Inquiry.

[41] Haverfield MC, Tierney A, Schwartz R, Bass MB, Brown-Johnson C, Zionts DL, Safaeinili N, Fischer M, Shaw JG, Thadaney S, Piccininni G, Lorenz KA, Asch SM, Verghese A, Zulman DM (2020). Can patient-provider interpersonal interventions achieve the quadruple aim of healthcare? A systematic review. J Gen Intern Med.

[42] Aldaz BE, Treharne GJ, Knight RG, Conner TS, Perez D (2017). Oncology healthcare professionals' perspectives on the psychosocial support needs of cancer patients during oncology treatment. J Health Psychol.

[43] Boston P, Bruce A (2018). Palliative care nursing, technology, and therapeutic presence: are they reconcilable?. J Palliat Care.

[44] Thorne SE, Kuo M, Armstrong E, McPherson G, Harris SR, Hislop TG (2005). 'Being known': patients' perspectives of the dynamics of human connection in cancer care. Psychooncology.

[45] Grover C, Mackasey E, Cook E, Nurse H, Tremblay L, Clinician N, Loiselle CG (2018). Patient-reported care domains that enhance the experience of "being known" in an ambulatory cancer care centre. Can Oncol Nurs J.

[46] O’Grady C, Dahm MR, Roger P, Yates L (2013). Trust, talk and the dictaphone: tracing the discursive accomplishment of trust in a surgical consultation. Discourse Soc.

[47] Crawford T, Roger P, Candlin S (2017). Tracing the discursive development of rapport in intercultural nurse-patient interactions. Int J Appl Linguist.

[48] Komatsu H, Yagasaki K (2014). The power of nursing: guiding patients through a journey of uncertainty. Eur J Oncol Nurs.

[49] Radwin LE, Farquhar SL, Knowles MN, Virchick BG (2005). Cancer patients' descriptions of their nursing care. J Adv Nurs.

[50] Rchaidia L, Dierckx de Casterlé B, De Blaeser L, Gastmans C (2009). Cancer patients' perceptions of the good nurse: a literature review. Nurs Ethics.

[51] Goodchild L, Mulligan A, Shearing GE, Mueller T (2022). Clinician of the Future; a 2022 Report.

[52] Hammersley V, Donaghy E, Parker R, McNeilly H, Atherton H, Bikker A, Campbell J, McKinstry B (2019). Comparing the content and quality of video, telephone, and face-to-face consultations: a non-randomised, quasi-experimental, exploratory study in UK primary care. Br J Gen Pract.

[53] Sandelowski M (2000). Whatever happened to qualitative description?. Res Nurs Health.

[54] Sandelowski M (2010). What's in a name? Qualitative description revisited. Res Nurs Health.

[55] Koppel PD, De Gagne JC (2021). Exploring nurse and patient experiences of developing rapport during oncology ambulatory care videoconferencing visits: protocol for a qualitative study. JMIR Res Protoc.

[56] Elo S, Kyngäs H (2008). The qualitative content analysis process. J Adv Nurs.

[57] Sandelowski M (1995). Sample size in qualitative research. Res Nurs Health.

[58] Marshall C, Rossman GB (2016). Designing Qualitative Research. Sixth edition.

[59] Hsieh H, Shannon SE (2005). Three approaches to qualitative content analysis. Qual Health Res.

[60] Morse JM, Barrett M, Mayan M, Olson K, Spiers J (2016). Verification strategies for establishing reliability and validity in qualitative research. Int J Qual Methods.

[61] Elo S, Kääriäinen M, Kanste O, Pölkki T, Utriainen K, Kyngäs H (2014). Qualitative content analysis: a focus on trustworthiness. SAGE Open.

[62] Tracy SJ (2010). Qualitative quality: eight “big-tent” criteria for excellent qualitative research. Qual Inq.

[63] Tong A, Sainsbury P, Craig J (2007). Consolidated criteria for reporting qualitative research (COREQ): a 32-item checklist for interviews and focus groups. Int J Qual Health Care.

[64] Unhjem JV, Vatne S, Hem MH (2018). Transforming nurse-patient relationships-a qualitative study of nurse self-disclosure in mental health care. J Clin Nurs.

[65] Radwin L (2000). Oncology patients' perceptions of quality nursing care. Res Nurs Health.

[66] Loos N (2021). Nurse listening as perceived by patients: how to improve the patient experience, keep patients safe, and raise HCAHPS scores. J Nurs Adm.

[67] Prip A, Pii KH, Møller KA, Nielsen DL, Thorne SE, Jarden M (2019). Observations of the communication practices between nurses and patients in an oncology outpatient clinic. Eur J Oncol Nurs.

[68] McIlfatrick S, Sullivan K, McKenna H, Parahoo K (2007). Patients' experiences of having chemotherapy in a day hospital setting. J Adv Nurs.

[69] Fredrickson B, Barrett LF, Haviland JM (2016). Positivity resonance as a fresh, evidence-based perspective on an age-old topic. Handbook of Emotions. 4th ed.

[70] Street RL, Makoul G, Arora NK, Epstein RM (2009). How does communication heal? Pathways linking clinician-patient communication to health outcomes. Patient Educ Couns.

[71] Kelley JM, Kraft-Todd G, Schapira L, Kossowsky J, Riess H (2014). The influence of the patient-clinician relationship on healthcare outcomes: a systematic review and meta-analysis of randomized controlled trials. PLoS One.

[72] Elliott T, Tong I, Sheridan A, Lown BA (2020). Beyond convenience: patients' perceptions of physician interactional skills and compassion via telemedicine. Mayo Clin Proc Innov Qual Outcomes.

[73] Rose S, Hurwitz HM, Mercer MB, Hizlan S, Gali K, Yu P, Franke C, Martinez K, Stanton M, Faiman M, Rasmussen P, Boissy A (2021). Patient experience in virtual visits hinges on technology and the patient-clinician relationship: a large survey study with open-ended questions. J Med Internet Res.

[74] Schmidt KL, Gentry A, Monin JK, Courtney KL (2011). Demonstration of facial communication of emotion through telehospice videophone contact. Telemed J E Health.

[75] Bohannon LS, Herbert AM, Pelz JB, Rantanen EM (2013). Eye contact and video-mediated communication: a review. Displays.

[76] Gupta A, Harris S, Naina HV (2015). The impact of physician posture during oncology patient encounters. J Cancer Educ.

[77] Hibbert D, Mair FS, May CR, Boland A, O'Connor J, Capewell S, Angus RM (2004). Health professionals' responses to the introduction of a home telehealth service. J Telemed Telecare.

[78] Sävenstedt S, Zingmark K, Hydén L-C, Brulin C (2005). Establishing joint attention in remote talks with the elderly about health: a study of nurses' conversation with elderly persons in teleconsultations. Scand J Caring Sci.

[79] Hastings SN, Mahanna EP, Berkowitz TSZ, Smith VA, Choate AL, Hughes JM, Pavon J, Robinson K, Hendrix C, Van Houtven C, Gentry P, Rose C, Plassman BL, Potter G, Oddone E (2021). Video-enhanced care management for medically complex older adults with cognitive impairment. J Am Geriatr Soc.

[80] Sharma U, Clarke M (2014). Nurses' and community support workers' experience of telehealth: a longitudinal case study. BMC Health Serv Res.

[81] Oliver DP, Albright DL, Kruse RL, Wittenberg-Lyles E, Washington K, Demiris G (2014). Caregiver evaluation of the ACTIVE intervention: "it was like we were sitting at the table with everyone". Am J Hosp Palliat Care.

[82] Foster PH, Whitworth JM (2005). The role of nurses in telemedicine and child abuse. Comput Inform Nurs.

[83] Read Paul L, Salmon C, Sinnarajah A, Spice R (2019). Web-based videoconferencing for rural palliative care consultation with elderly patients at home. Support Care Cancer.

[84] Tachakra S, Rajani R (2002). Social presence in telemedicine. J Telemed Telecare.

[85] Christensen LF, Wilson R, Hansen JP, Nielsen CT, Gildberg FA (2021). A qualitative study of patients' and providers' experiences with the use of videoconferences by older adults with depression. Int J Ment Health Nurs.

[86] Draper JV, Kaber DB, Usher JM (1998). Telepresence. Hum Factors.

[87] Turner K, Bobonis Babilonia M, Naso C, Nguyen O, Gonzalez BD, Oswald LB, Robinson E, Elston Lafata J, Ferguson RJ, Alishahi Tabriz A, Patel KB, Hallanger-Johnson J, Aldawoodi N, Hong Y, Jim HSL, Spiess PE (2022). Health care providers' and professionals' experiences with telehealth oncology implementation during the COVID-19 pandemic: a qualitative study. J Med Internet Res.

[88] Darcourt JG, Aparicio K, Dorsey PM, Ensor JE, Zsigmond EM, Wong ST, Ezeana CF, Puppala M, Heyne KE, Geyer CE, Phillips RA, Schwartz RL, Chang JC (2021). Analysis of the implementation of telehealth visits for care of patients with cancer in Houston during the COVID-19 pandemic. JCO Oncol Pract.

[89] Lindberg B, Axelsson K, Öhrling K (2009). Taking care of their baby at home but with nursing staff as support: the use of videoconferencing in providing neonatal support to parents of preterm infants. J Neonatal Nurs.

[90] Perry A, Federico F, Huebner J (2021). Telemedicine: Ensuring Safe, Equitable, Person-Centered Virtual Care.

[91] Binder AF, Handley NR, Wilde L, Palmisiano N, Lopez AM (2020). Treating hematologic malignancies during a pandemic: utilizing telehealth and digital technology to optimize care. Front Oncol.

[92] Myronuk L (2022). Effect of telemedicine via videoconference on provider fatigue and empathy: implications for the Quadruple Aim. Healthc Manage Forum.

[93] Chike-Harris KE, Garber K, Derouin A (2021). Telehealth educational resources for graduate nurse faculty. Nurse Educ.

[94] Allen Watts K, Malone E, Dionne-Odom JN, McCammon S, Currie E, Hicks J, Tucker RO, Wallace E, Elk R, Bakitas M (2021). Can you hear me now?: improving palliative care access through telehealth. Res Nurs Health.

[95] Cheng C, Humphreys H, Kane B (2021). Transition to telehealth: engaging medical students in telemedicine healthcare delivery. Ir J Med Sci.

[96] Nagel DA, Penner JL (2016). Conceptualizing telehealth in nursing practice: advancing a conceptual model to fill a virtual gap. J Holist Nurs.

[97] Haertl KL, Theismann A (2022). Perceived impact of a relationship-based care curriculum. Creat Nurs.

[98] Reilly J, Krause K, Vande Zande C, Knutzen B (2019). Implementing relationship-based care as a professional practice model: promoting nurses' understanding and confidence to apply in practice. Creat Nurs.

[99] Zulman DM, Haverfield MC, Shaw JG, Brown-Johnson CG, Schwartz R, Tierney AA, Zionts DL, Safaeinili N, Fischer M, Thadaney Israni S, Asch SM, Verghese A (2020). Practices to foster physician presence and connection with patients in the clinical encounter. JAMA.

[100] Ansari N, Wilson CM, Heneghan MB, Supiano K, Mooney K (2022). How technology can improve communication and health outcomes in patients with advanced cancer: an integrative review. Support Care Cancer.

[101] Sieck CJ, Rastetter M, Hefner JL, Glover AR, Magaña C, Gray DM, Joseph JJ, Panchal B, Olayiwola JN (2021). The five A's of access for TechQuity. J Health Care Poor Underserved.

[102] Rivera V, Aldridge MD, Ornstein K, Moody KA, Chun A (2021). RESEARCHRacial and socioeconomic disparities in access to telehealth. J Am Geriatr Soc.

[103] Benda NC, Veinot TC, Sieck CJ, Ancker JS (2020). Broadband internet access is a social determinant of health!. Am J Public Health.

[104] Zahnd WE, Bell N, Larson AE (2022). Geographic, racial/ethnic, and socioeconomic inequities in broadband access. J Rural Health.

[105] Majeed A, Maile E, Coronini-Cronberg S Covid-19 Is Magnifying the Digital Divide.

[106] National Cancer Institute at the National Institutes of Health Cancer Disparities.

[107] Vohra J, Marmot MG, Bauld L, Hiatt RA (2016). Socioeconomic position in childhood and cancer in adulthood: a rapid-review. J Epidemiol Community Health.

