# Supplementary material for: Exploring Nurse and Patient Experiences of Developing Rapport During Oncology Ambulatory Care Videoconferencing Visits: Qualitative Descriptive Study
Source: J Med Internet Res. 2022 Sep 8;24(9):e39920. doi: 10.2196/39920 (PMC9501656; doi:10.2196/39920)
Supplement: Multimedia Appendix 1 [file jmir_v24i9e39920_app1.docx]

Multimedia Appendix 1

Sample characteristics of persons with cancer (N=10)

| Characteristic | Mean (SD, range)/Frequency (%) |
| --- | --- |
| Age (years), mean (range) | 54.3 (8.31, 36-67) |
| Gender  Female  Male | 7 (70)  3 (30) |
| Ethnicity/race  White/Caucasian  All other ethnic/racial categories | 10 (100)  0 (0) |
| Education  Less than high school diploma  Some college or associate’s degree  Bachelor’s degree  Graduate or professional degree | 0 (0)  3 (30)  2 (20)  5 (50) |
| Marital status  Married | 7 (70) |
| Employment status  Full-time  Part-time  Seeking employment  Retired | 7 (70)  2 (20)  1 (10)  0 (0) |
| Household income (US dollars)^a^  Less than $25,000  $25,000-49,999  $50,000-74,999  $75,000-99,000  $100,000-149,999  $150,000 or greater | 0 (0)  1 (10)  1 (10)  1 (10)  2 (20)  4 (40) |
| Time in treatment (years)  Less than one year  1-3 years  More than 3 years | 5 (50)  3 (30)  2 (20) |
| Video visits past 12 months, mean (range)  1-2 videoconference visits  3-5 videoconference visits  More than 5 videoconference visits | 5.15 (3.51, 2-12)  1 (10)  7 (70)  2 (20) |
| Video visits past 3 months  Yes  No | 7 (70)  3 (30) |
| Time per day on computer (hours)  Less than one  1-3  4-6  More than 7 | 0 (0)  3 (30)  4 (40)  3 (30) |
| Personal/work videoconference meetings past year  Less than 10  10-49  50-100  Over 100 | 2 (20)  3 (30)  2 (20)  3 (30) |

SD: Standard deviation

^a^One participant declined to answer this question
