# Supplementary material for: Exploring Nurse and Patient Experiences of Developing Rapport During Oncology Ambulatory Care Videoconferencing Visits: Qualitative Descriptive Study
Source: J Med Internet Res. 2022 Sep 8;24(9):e39920. doi: 10.2196/39920 (PMC9501656; doi:10.2196/39920)
Supplement: Multimedia Appendix 2 [file jmir_v24i9e39920_app2.docx]

Multimedia Appendix 2

Sample characteristics of oncology nurses (N=12)

| Characteristic | Mean (SD, range)/Frequency (%) |
| --- | --- |
| Age (years), mean (SD, range)  Less than 30 years old  31-40 years old  41-50 years old  51-60 years old | 42.25 (8.62, 32-59)  0 (0)  5 (41.6)  5 (41.6)  2 (16.7) |
| Gender  Female  Male | 10 (83.3)  2 (16.7) |
| Ethnicity/race^b^  White/Caucasian  African American-Other  All other ethnic/racial categories | 11 (91.7)  1(8.3)  0 (0) |
| Nursing education  Diploma  Associate’s degree  Bachelor’s degree  Master’s degree  Doctorate degree | 1 (8.3)  0 (0)  2 (16.7)  8 (66.7)  1 (8.3) |
| Nursing experience (years)  Less than 5 years  5-10 years  11-20 years  21-30 years  Over 31 years | 16.54 (8.67, 4-35)  2 (16.7)  1 (8.3)  5 (41.6)  3 (25)  1 (8.3) |
| Cancer center employment (years)  Less than 2 years  2-5 years  6-10 years  11-20 years  Over 20 years | 10.5 (9.06, 1-33)  1 (8.3)  3 (25)  2 (16.7)  4 (33.3)  2 (16.7) |
| Video visits past 3 months  Less than 15 visits  15-50 visits  51-100 visits  More than 100 visits | 43.42 (63.06, 3-240)  5 (41.7)  4 (33.3)  2 (16.7)  1 (8.3) |
| Video visits past 12 months,  Less than 15 visits  16-60 visits  61-100 visits  101-200 visits  More than 200 visits | 168.17 (247.37, 3-960)  1 (8.3)  4 (33.3)  1 (8.3)  5 (41.7)  1 (8.3) |

SD: Standard deviation

^b^One participant listed ethnicity as African American and other
