# Supplementary material for: Exploring Nurse and Patient Experiences of Developing Rapport During Oncology Ambulatory Care Videoconferencing Visits: Qualitative Descriptive Study
Source: J Med Internet Res. 2022 Sep 8;24(9):e39920. doi: 10.2196/39920 (PMC9501656; doi:10.2196/39920)
Supplement: Multimedia Appendix 3 [file jmir_v24i9e39920_app3.docx]

Multimedia Appendix 3

Data Analysis of Patient Participant Interviews

**Overarching Theme: Patients view VCV with their health care clinicians positively and appreciate having a personal relationship with their nurse, which they find is achievable in VCVs.**

| **Theme 1: Building rapport in VCVs and IPVs requires a personal touch.** | | |
| --- | --- | --- |
| Categories | Codes | Exemplars |
| Feeling known as a person not a patient | - active listening - personalized care - small talk to build rapport - feels more personal - treated as a person not patient - finding a common thread - self-disclosure | [Videoconferencing visits are] certainly awkward in the beginning. You're meeting a stranger face to face. But thinking back to my experience, the nurse asks me some personal questions about myself to get me talking about myself. I think that helped …break down some of the barriers, just makes you feel more comfortable. [P09]  Developing that connection is understanding somebody as a person, and it's harder than in person because I don't get to know what their shoes are like. I don't really see the whole person, so having those personal touches, having that chitchat at the beginning, they're sort of more important than they are in the in-person visits, and they're in some ways harder to do. [P10]  I feel that creating that human connection through asking personal questions is a way to start to establish rapport. [P06] |
| Being heard and knowing what’s important | - active listening - being heard and understood - empathy and compassion - knowing the patient - knowing we are cared for - feeling I’m important - willingness to share - knowing what’s important - emotional comfort level | I'm not just my cancer diagnosis, and a lot of the things that matter to me about my cancer diagnosis have very little to do with what's growing in my body and everything to do with how it impacts the things that are important to me. [P10]  [Rapport] is very important to me. I think when I have rapport with someone, I really feel like they understand what I'm saying. I feel validated. I feel like I'm important in the conversation,...I'm worth having an appointment with. [P03] |
| Taking time to provide information and answer questions thoughtfully | - no question is too silly - taking time - empathy and compassion - knowing we are cared for | It was my asking questions and not feeling that any of the questions were stupid or silly or anything like that, that they were open to whatever information I needed. [P02]  I think it's encouraged by the nurse. When nurses ask open-ended questions, it encourages a dialogue. It makes you feel like they care, and they really want to know. They're not just asking yes or no questions. [P09] |
| **Theme 2: Rapport can facilitate trust in videoconferencing and impact how patients feel about their care.** | | |
| Creating an open atmosphere with positive affect and nonverbal communication | - friendliness and positive affect - humanizing the virtual box - eye contact - facial expressions - voice tone and clarity - body language - compensating for lack of physical presence | Having good eye contact, actually speaking positively…being friendly, making sure that you're engaged in what you're saying and who you're talking to. I think sometimes you can hear it in the voice,...making sure...you look at the person, that you're not looking away. [P03]  If the nurse’s facial expression and demeanor show the concern and care [for] the patient, it’s more apt to work. [P02] |
| Building confidence in the plan of care | - confidence in provider - credibility as expert - being on the same page - improves quality of care - relationship with mutual understanding - emotional comfort level | It's very important, the rapport, to have that level of trust and…comfort with your nurses,…[to] rely on them giving you a truthful, honest answer, or even if they don't know a definite answer [to know] that they're…giving you the whole picture. [P08]  The more that the nurse [understands what’s important to me], the easier it is for them to actually make the right treatment decisions…quality of life is [more] important to me…than the quantity, and so that has implications. [P10]  What I really liked about [the cancer center] was you had the same nurse primarily through your entire treatment...We talked about various different things...that trust factor from them, the familiarity with them, and understanding that they're really here to care for you. [P05]  I guess a big thing is that I want to feel comfortable that I can ask as many questions as I need to, that there isn't a rush on the time, and that once I ask the question, I'm not just getting a pat answer, it's customized to me or to my situation…it's not the person just getting their job done, but they're really trying to help me. [P06] |
| Promoting comfort and healing | - mutual trust - promotes healing - trust as outcome of rapport - a valued relationship - having the same nurse - mutual care and concern - emotional comfort level | This is a difficult journey. You're going through stuff. So much is just unknown to you, and one day you're going on in your life, and then the next day you have cancer.  It's very, very important, the rapport, because to have that level of trust and have that level of comfort with your nurses, whether it's over video or in person...it's so important because you're going through this. [P08] |
| **Theme 3: Videoconferencing works well for some visits but is not ideal for others.** | | |
| Leveraging the advantages of VCV and technology | - easier - efficient - more options - more relaxed when I’m at home - saves energy for getting better - love it - being on time | I find it much more convenient than driving to [the cancer center]…an hour each way. [P07]  It makes it so that the massive number of doctors' appointments is a little less overwhelming to my ability to do things like be there for my family or have a job. [P10]  There were times when [the nurse] was like, "Don't come in here. There's no reason for you to come in here. Let's do a telehealth, and don't waste the time. You need to focus on your treatment and getting better." [P05] |
| Accepting the challenges of a new technology | - not feasible in VCV - harder to share bad news - Something is lost in VCV - figuring it out - determining what’s best - combination may be best - willingness to compromise - internet connectivity - avoiding distractions | My generation keeps on getting older, so there’s going to be a higher percentage of patients [at the cancer center] that are going to be technology savvy. [P04]    Sometimes it's things you can't anticipate. The internet goes out. Your power goes out, those things can happen…to anybody at any time. [P08]  I want to say I prefer in-person meetings to virtual meetings. I can't put my finger on it because I feel like it's more about the people and the exchange than it is about doing it through the computer. Something seems more personal when you're in person, but I don't know that it's actually that different except for where a nurse would need to examine you. [P06] |
| Finding minimal differences in terms of communication effectiveness | - no different than in person - bulk of communication can be done in VCV | There was really nothing discussed that couldn't be discussed [in a VCV]…I was there anyway, and he was there anyway, and it [VCV] gives you the old home feeling of face to face, but that to me is obsolete now...the only support would be drilling deeper into an answer I might give, still that would occur [in a VCV]. [P07]  I find it very easy to ask questions. I don't find it challenging to express my emotions over videoconferencing. I've had to say, "I don't like this," or "Can you clarify this point to me?" I don't find it difficult to either express feelings or ask questions to the nurses around any problems or concerns...so I don't find it to be different. [P04] |
