# Supplementary material for: Exploring Nurse and Patient Experiences of Developing Rapport During Oncology Ambulatory Care Videoconferencing Visits: Qualitative Descriptive Study
Source: J Med Internet Res. 2022 Sep 8;24(9):e39920. doi: 10.2196/39920 (PMC9501656; doi:10.2196/39920)
Supplement: Multimedia Appendix 4 [file jmir_v24i9e39920_app4.docx]

Multimedia Appendix 4

Data Analysis of Nurse Participant Interviews

**Overarching Theme: Rapport develops when nurses know their patients as persons and is achievable in VCVs with some adaptations.**

| **Theme 1: Rapport building begins with nurses knowing their patients as persons** | | |
| --- | --- | --- |
| Categories | Codes | Exemplars |
| Viewing the patient from a holistic perspective | - holistic knowing - personalized care - feels more personal - treated as a person not patient - knowing the patient | It's huge to understand and see a patient...holistically and for them to feel comfortable, supported and positively regarded,...to take care of them as a person, not always just a patient, and for them to feel like we're interested in their lives, and we want the best for them. [N05]  We really get an opportunity to meet our patients, to get to know them as individuals, to really get to know who they are as persons, not just their illness....[W]hat helps patients feel well cared for is that sense of rapport, that sense of being cared for as a human being…and that's the thing that people will remember. [N09]  People are different….It’s really at their comfort level. Sometimes they’d rather keep it to the task at hand. But I want to at least allow an opportunity for them to share more if they’re willing. [N12] |
| Attentiveness and listening to know the patient and understand their perspective | - active listening - being heard and understood - empathy and compassion | What is important to them but…might scare them, and what their concerns are, is a huge aspect of almost every conversation I have with a patient. Their biggest concerns, their biggest worries, and oftentimes those worries, they carry through into future visits. [N05] |
| Shared experiences enhance trust, open communication, and quality of care | - finding a common thread - shared experience - willingness to share - trust - improved quality of care - enhanced clinical judgement | It has to go both ways. You can't just expect information and not give it, and you build a better relationship when you give a little bit of yourself. I don't give anything too revealing, but if it's a patient that's similar in age to me, we'll talk about our kids, light stuff...I try to find some common thread that we can sort of bond over, and I do think that's really important in my practice. I make a point of doing that. [N04]  Trying to relate is the best thing. Finding a common topic of interest, a common ground...it's impossible not to have some type of connection. Even if there's a complete difference,…the commonality is that we're different. It's just establishing a connection. I mean, if you have good rapport with someone, they're able to open up to you more. If you have bad rapport with someone, they're probably a little more guarded...so of course, better rapport, better care. [N12] |
| **Theme 2: Much of bedside manner can be translated into “webside” manner.** | | |
| Ability to communicate similar to in person | - bulk of communication can be done in VCV - no different than in person | The context of our conversations pretty much unfolded in very similar ways in person as well as over videoconferencing. It's a very conversational, open, free-flowing type of two-way street....With video visits, that ability was not changed or affected negatively at all…they had all the time they needed to unpack that stuff. [N05] |
| Making extra efforts to connect | - friendliness and positive affect - small talk - humanizing the virtual box - eye contact - body language - compensating for lack of physical presence | The human qualities that we all have are better delivered in person, and we try to have different inflections in your voice when you're just on video because you want them [patients] to understand that you do hear and you do feel what emotion they're trying to share with you, but otherwise it could be just like watching, the same news story over and over again. [N08]  I try to be a good listener. Silence is a good thing. It allows patients to think about what they want to say, and I'm just there with them in the moment. You can do that on video and on an in-person visit.…Being able to see and just be there with the patient and know that they're still looking at you, and I'm still looking at them. [N07] |
| Ability to form relationships and develop rapport remain intact | - no different than in in-person experience - relationship valued by patient and nurse | For the majority of my patients, [clinical rapport in VCVs and IPVs is] pretty similar….[W]e get down to the medical stuff and teaching and side effects and medication management. But with a lot of patients, it's more of a social rapport. Especially patients that we see frequently or that I've known for a long time. They can talk about their family. They're asking about my family, and I don't see a difference with video versus in person. [N07]  I usually try to be very welcoming. I use a lot of comedy. I like humor, so I try to endear myself to them through, kind of mechanisms like that, but I don't feel like a lot is lost in that regard, at least with me. [N11] |
| **Theme 3: Differences in VCV that may impact rapport are important to recognize.** | | |
| There is only so much you can do on Zoom | - not feasible - loss of emotional connect - more information when physically in same space - knowing something is not right - something is lost in VCV - reading the room - harder to do - harder to share bad news | You really only have your own facial expression, and your volume, and your tone, and your words when it's done by video, but all of the rest of the human connection is different in person. [N08]  It's a different level of attention when you do something on the video…a person walking through a door of an exam room is just such a wealth of opportunity to draw upon in a visit. And you just lose a lot of that when things are done virtually. [N11] |
| There’s no place like home | - more relaxed at home - easier for patients - even playing field - safer during COVID-19 | I thought that it was really lovely to see people in their own homes, and also people seemed so much more relaxed because it was like on their turf. There was almost [more] control.…[W]hen people come into the hospital, they sit in traffic, they sit in the waiting room, they get this tag, and they're kind of at the mercy of the provider. At home, they can be in their pajamas. They can have their cup of coffee. They don't have to wear a mask. [N09] |
| Barriers related to technology, language, and hearing | - language/cultural differences - technology issues - hearing deficits | Language barriers. We've done it, when we have an interpreter, and the patient, and the Zoom, it can work, but it definitely is adding in a third person....Everyone kind of has to be ready at the same time. [N09]  I have some patients [who] can't get onto the video visit no matter how hard we try, and sometimes those become a telephone visit, but when we can get everybody to use it, it's [still] not perfect. It's very good, but it's not perfect. [N08] |
| **Theme 4: Cultivating nurse-patient rapport in VCVs is essential for quality care and nurse job satisfaction.** | | |
| Everyone is still learning, but we are finding ways to adapt | - figuring it out - strategies to enhance rapport in VCVs | We're all still learning, the patients have been wonderful….This is still all new to them as well, so we're all in the same situation, so I feel like everybody's understanding. [N06] |
| Determining what is best for the person and the patient | - IPVs preferable - combination best - patient preference - willingness to compromise for VCV benefits - easier for patients | I mean there's definitely advantages to being in person,…I'm not sure that that outweighs the benefit of the televisit. [N09]  There are certain patients [who] maybe respond better in person, and maybe our patients [who] are not as tech-savvy, they still need to be seen in person. But our patients [who] feel very comfortable with technology, they've been doing this for two years, it just is so extremely helpful in terms of people's personal lives. [N10]  There may be a slight advantage to having [emotional] conversations in person and being able to physically touch the patient...[to] show our emotion and compassion versus just facial expression. [N03] |
| The unique challenges presented by VCVs require professional development and practice guidelines | - figuring it out - strategies to enhance rapport in VCVs | Figuring out the patients who would be appropriate is the harder part. [N02]  It's important to have expectations in place…even though it is a good substitute, it cannot completely or should not, in my opinion, completely replace [IPVs]. [N05] |
| Developing rapport with patients is important to oncology nurses | - relationship valued by patient and nurse - intimate connection - impact on stress - it makes it fulfilling | [Rapport is] why I've done my job for as long as I have. I've been here for 20 years in the same group and being able to develop rapport and get to know patients, get to know their families and be with them from diagnosis to death, is what keeps me coming back every day. [N07]  When you foster and build a successful rapport with your patient, then they ask questions more easily, they report symptoms more easily...I have some patients [who] insisted if they were doing a virtual video visit with their physician, I had to also be on the visit, and I really appreciated them valuing my role on their team. [N08] |
